# Geographic mobility among older people and their adult children: The role of parents' health issues and family ties

**DOI:** 10.1002/psp.2371

**Published:** 2020-08-11

**Authors:** Alyona Artamonova, Brian Joseph Gillespie, Maria Brandén

**Affiliations:** ^1^ Population Research Centre, Faculty of Spatial Sciences University of Groningen Groningen The Netherlands; ^2^ Demography Unit Stockholm University Stockholm Sweden; ^3^ The Institute for Analytical Sociology Linköping University Norrköping Sweden

**Keywords:** gender, health problems, intergenerational geographic proximity, internal migration, population register data, Sweden

## Abstract

This research examines the relationship between older parents' health issues and (i) their relocations closer to their faraway adult children, (ii) their relocations into institutionalised care facilities, or (iii) having distant children move closer. Additionally, we investigate how these relocations are structured by children's gender and location. We focused on parents aged 80 years and older and their distant children. Multinomial logistic regression models were employed for older men and women based on data from administrative registers of Sweden. Whereas severe health problems were associated with an increased likelihood of parent relocations closer to their children or into institutions, they were not associated with the likelihood of children's moves towards parents. Mothers were more likely to move towards daughters or towards distant children who had at least one sibling living nearby. Children moved closer to their parents when there was at least one sibling living near the parent or in response to their own life circumstances.

## 1 INTRODUCTION

Population ageing means that societies must increasingly adapt to older adults' needs for personal and practical care. Age‐related vulnerabilities and health problems can motivate people to turn to public services or kin support networks for assistance. Although caring for relatives is not a legal obligation in many European countries because the state usually takes this responsibility, family remains an important source of support for older people (Künemund & Rein, 1999). The availability, regularity, and amount of this support is facilitated by geographic proximity between family members (Dewit, Wister, & Burch, 1988; Lawton, Silverstein, & Bengtson, 1994). However, current population ageing and welfare‐state retrenchment might increase the dependence of older parents on their adult children, especially daughters who are more likely to be in contact with elderly parents at later stages of their lives (Grigoryeva, 2017; Lennartsson, Silverstein, & Fritzell, 2010). Parents who live close to their children can usually rely on them, whereas long distances might motivate parents and/or children to move closer to each other. Another option for older parents dealing with health problems is to utilise support from the state or private market, including institutionalised residential care.

Previous studies point to associations between parents' and their adult children's life course events, such as marital separation, widowhood, and childbirth and related support needs, on one hand, and the likelihood of intergenerational proximity‐enhancing moves, on the other (Pettersson & Malmberg, 2009; Thomas & Dommermuth, 2020; Zhang, Engelman, & Agree, 2013). These moves improve family members' opportunities to care for each other (Vergauwen & Mortelmans, 2020). Some studies also suggest that clustering of family members (e.g., other children, siblings, and grandchildren) reinforces their attracting effects for migration (Pettersson & Malmberg, 2009; Thomas & Dommermuth, 2020). At the same time, living near family members has a strong migration‐deterring effect (Ermisch & Mulder, 2019; Hünteler & Mulder, 2020).

Most research on the topic has separately considered the role of parents' and children's needs or the location of other family members for the relocation behaviour of parents and adult children but rarely both at the same time. It is therefore unclear how parents and their distant children respond to needs for care at the final stage of parents' lives, when their health deteriorates and they transition to dependency. As such, the central questions of this study are as follows: *How are parents' health problems later in life associated with (i) relocations closer to faraway adult children, (ii) into institutionalised residential care, or (iii) having their distant children move nearby? And, how are these relocations patterned by adult children's gender and location?* To address these questions, we use multinomial logistic regression to analyse population register data from Sweden between 2013 and 2017. We control for location‐specific capital of parents and children, their sociodemographic characteristics, and the degree of urbanisation of their place of residence.

## 2 THEORETICAL AND EMPIRICAL BACKGROUND AND HYPOTHESES

The developments in health and social policy during the 20th century contributed to an extended lifespan and increased the quality of human ageing (Gilleard & Higgs, 2010). Recently, researchers have conceptualised the period of “later life” as being divided into two phases: the young‐old and oldest‐old (Smith, 2001). The first phase, also referred to as the “third age,” relates to postretirement opportunities and personal fulfilment, whereas the second is linked to illness, dependence, and death (Baltes & Smith, 2003; Higgs & Gilleard, 2015; Laslett, 1991). This “fourth age” is marked by a diminished capacity for self‐care and difficulties in performing daily chores and social exchange—this group is the focus of the current study (Lloyd, Calnan, Cameron, Seymour, & Smith, 2014).

Research indicates that the time of transition from the third to the fourth age generally occurs between ages 80 and 85 years (Blanchard‐Fields & Kalinauskas, 2009). Individuals in this age group have a higher propensity to experience losses in vision, hearing, strength, functional capacity, psychological adaptivity, and cognitive functions, as well as losses in happiness and social contacts (Baltes & Smith, 2003; Smith, 2001). In the transition to the fourth age, gender differences apply: women often endure multiple chronic disabilities and degenerative illnesses, such as arthritis, high blood pressure, and hearing impairment, whereas men are more likely to suffer from fatal conditions, such as heart disease and cancer (Leveille, Resnick, & Balfour, 2000). Although women live longer than men on average, their health and quality of life is often poor in their later years (Solé‐Auró, Jasilionis, Li, & Oksuzyan, 2018).

In the circumstances of diminished health, the oldest‐old, in general, tend to lose their independence and require advanced care (Speare, Avery, & Lawton, 1991; Worobey & Angel, 1990). In most developed countries, older people can receive necessary assistance from formal (public care) and informal (family members, friends) sources (Connidis & Barnett, 2018). Care responsibilities can rest on the welfare state or private market, whereby an older person in the fourth age might transition to institutionalised care or receive in‐home care. According to Denton (1997), formal care usually compensates for the absence of informal caregivers and, in some cases, supplements the assistance of the spouse or adult child caregiver for personal care tasks and housework.

Often, for frail older men and women, the family is an essential source of care, which includes both practical and emotional support (Brody, 1981; Lloyd et al., 2014). The most likely providers of health‐related care are spouses and children (Connidis & Barnett, 2018). The closer children live to parents (i.e., the shorter they must travel to the parent), the more support they provide (Dewit et al., 1988; Kalmijn & Dykstra, 2006).

The intergenerational geographic proximity needed for extensive support exchange develops throughout a long period of time, and its importance can vary at different stages of parents' and children's life courses. According to Lin and Rogerson's three‐stage developmental model of intergenerational proximity and life‐cycle mobility (Lin & Rogerson, 1995), the first intergenerational spatial separation takes place when adult children leave the parental home for education, employment, and/or marriage. The second stage represents a stabilisation of intergenerational proximity, resulting from adult children's life‐cycle migration. This can take them further away from parents (e.g., in search of better job opportunities) or bring them closer (e.g., if help with grandchildren is needed). In the final stage, parents' increasing need for health care—sometimes compounded by losing a partner—can lead to proximity‐enhancing moves made by parents or children, also known as *geographic convergence* (Silverstein, 1995). This final stage corresponds with the second and third stages of Litwak and Longino's classic model of geographic mobility in later life, whereby older parents move to the vicinity of adult children as their potential caregivers or to residential care facilities (Litwak & Longino, 1987). Older people in the fourth age are likely to be in greater need of health‐related family care, and, therefore, of close intergenerational distance, than the young‐old.

Empirical studies from different countries have documented the relationship between older people's needs for care and residential adjustments (Golant, 2011; Zimmerman, Jackson, Longino, & Bradsher, 1993), including relocation closer to children and having adult children moving closer (Choi, 1996; Choi, Schoeni, Langa, & Heisler, 2014). Notably, when parents and children move close to each other, the person in need of care is more likely to move (Smits, 2010). On the basis of these insights, we propose two hypotheses related to health of an older parent: parents will be more likely to relocate closer to a child or have a child move closer when parents have severe health problems than when they are in better health (*Hypothesis 1a*) but the effect of health issues will be stronger for moving closer to a child than for having a child move closer (*Hypothesis 1b*). However, it is worth bearing in mind that some parents in need of care might have moved closer or had a distant child move closer prior to our observation period. This selection mechanism might supress the strength of the expected effects (Michielin, Mulder, & Zorlu, 2008).

Growing older can mean receiving less support from informal sources (Cranswick, 2003). Accordingly, when health continues to decline and older parents develop more severe health problems, they are likely to move to institutionalised residential care (Litwak & Longino, 1987; Van der Pers, Kibele, & Mulder, 2015). Therefore, we expect the effect of severe health problems to be stronger for relocation to institutionalised care than for geographic convergence (*Hypothesis 1c*).

In addition to parental health, another factor that might impact the probability of geographic convergence is the child's potential to provide support. Compared with sons, daughters generally provide more care, and more support with activities of daily living, in particular (Crawford, Bond, & Balshaw, 1994; Haberkern, Schmid, & Szydlik, 2015; Silverstein, Gans, & Yang, 2006). Research findings indicate that older parents are more likely to expect to move closer to daughters than to sons (Silverstein & Angelelli, 1998), and greater parental functional impairment decreases the selection of sons even more. Moreover, in Sweden, men live closer to their parents than women (Malmberg & Pettersson, 2007), who tend to move longer distances towards their partner (Brandén & Haandrikman, 2019). Thus, daughters or their older parents might move closer to each other more frequently when parental needs for health‐related care are high. Another study of caregiver selection among children pointed to an overrepresentation of daughters in relocations for their mother's care only (Leopold, Raab, & Engelhardt, 2014). We, therefore, hypothesise that all else equal, the propensity to migrate towards daughters will be greater than the propensity to move towards sons (*Hypothesis 2a*) and the propensity to have daughters moving closer will be greater than the propensity to have sons moving closer (*Hypothesis 2b*). Relatedly, these effects will be present only for older mothers (*Hypothesis 2c*).

Based on a family ties perspective (Mulder, 2018), nonresident family living nearby are a type of location‐specific capital that deters relocation (DaVanzo, 1981). Other children living close to parents (denoted from here as *parents' family ties*) might affect the likelihood of geographic convergence or parents' institutionalisation, not least because these (geographically close) children might be able to provide a parent with needed care. Older people with children living very close tend to change residence (Spring, Ackert, Crowder, & South, 2017) and utilise institutionalised care less often (Van der Pers et al., 2015) than those whose closest children live far away.

Therefore, we expect that older people who have other children living nearby will be less likely to move towards their distant child (*Hypothesis 3a*) or become institutionalised (*Hypothesis 3b*) than those who do not have other children living in close proximity. Studies also suggest that having siblings living close to parents form an additional attraction for relocation of their brothers and sisters (Michielin et al., 2008; Pettersson & Malmberg, 2009; Thomas & Dommermuth, 2020). Building on this insight, we hypothesise that having other children nearby will be associated with a higher likelihood of a distant child moving closer (*Hypothesis 3c*).

The presence of other children nearby the distant child (denoted from here as *children's family ties*) might also affect the likelihood of intergenerational geographic convergence. On one hand, moving towards the location of several children rather than one child can be more beneficial for parents, because they might rely on support of multiple informal caregivers in this case. On the other hand, distant children with a sibling living nearby might be less likely to move away because that sibling can also be thought of as a form of location‐specific capital. A Norwegian study found a positive relationship between adult children's family ties and the propensity of convergence (Thomas & Dommermuth, 2020). Thus, we hypothesise: older people who have at least two distant children clustered in one location will be more likely to move closer to them (*Hypothesis 4a*) and less likely to have at least one of these distant children moving closer (*Hypothesis 4b*) than those who have a distant child with no siblings living nearby.

Of course, relocation decisions of older parents and their adult children might be linked to other determinants not directly related to parental need or family ties. Being older is usually linked to a lower likelihood of migration (Bernard, Bell, & Charles‐Edwards, 2014) with the exception of oldest‐old people, for whom increasing age is associated with a slight uptick in migration propensity (Litwak & Longino, 1987). In general, people who are settled and have location‐specific capital in the area are more likely to stay (Fischer & Malmberg, 2001). Being married, having children, being employed, and length of residence are also conditions that restrain migration. For older people, coresidential partners might deter relocation and the utilisation of institutionalised care (Greene & Monahan, 1987; Larsson & Thorslund, 2002; Van der Pers et al., 2015), because partners are an important resource of help in later life (Messeri, Silverstein, & Litwak, 1993). The presence of dependent children in the adult children's household decreases the likelihood of relocation, but if the dependent children are at the preschool age, the likelihood of migration towards older parents might increase (Thomas & Dommermuth, 2020).

Dwelling size is a reflection of housing quality and also an indicator of the ability of parents or adult children to offer the necessary space for moving in together. Additionally, older adults (especially single ones) might downsize when they live in big houses because smaller dwellings are more manageable (Abramsson & Andersson, 2016). People with higher educational attainment and income are more likely to move (Chiswick, 2000) and more likely to do so irrespective of the location of family members (Silverstein, 1995) than others. Having fewer financial resources, in turn, is associated with closer geographic proximity between older parents and adult children (Silverstein, 1995).

Intergenerational distances are usually shorter for people with an immigrant background, especially those coming from low‐income countries, than for those born in Sweden (Malmberg & Pettersson, 2007). Additionally, immigrant parents are more likely than native parents to move closer to their children or have children migrating into coresidence (Thomas & Dommermuth, 2020). Adult children might be more willing to stay in—or move to—urban areas because they have more dynamic labour markets, whereas very old people might aim for cities because of better access to public services and formal care facilities compared with rural areas (Stockdale & Catney, 2014).

Sweden provides an interesting social context for this study. With one of the longest life expectancies in Europe and a strong defamilisation policy, the country is often considered an individualistic one. The country is also known for the older population's dependence on the high‐quality welfare services, including institutionalised care systems, rather than relying on kin support (Svallfors, 2004). However, public‐sector‐based assistance to the elderly has become less universal, more modest, and family‐oriented over the three past decades (Edebalk & Petersson, 2000; Ulmanen & Szebehely, 2015). The current Swedish eldercare system aims to provide older citizens with the opportunity for ageing at home for as long as possible. Families are encouraged to support their older family members in order to complement formal care services. Researchers report that contacts between adult children and their older parents are frequent (Daatland & Lowenstein, 2005; Ogg & Renaul, 2006), and informal care, especially provided by middle‐aged daughters (Szebehely, & Ulmanen, 2009) and children who live nearby (Johansson, Sundström, & Hassing, 2003), is high. Despite these changes, Sweden still has a relatively weak tradition of intergenerational care, one of the lowest propensities of people moving closer to family (Vergauwen & Mortelmans, 2020), and one of the strongest formal care provision policies in Europe (Haberkern & Szydlik, 2010). For these reasons, we expect high rates of institutional care utilisation, modest propensities of intergenerational geographic convergence due to parents' needs, and relatively weak effects of family ties on relocations within the country.

## 3 DATA AND METHODS

### 3.1 Data selection

Data from several Swedish population and administrative registers were combined to capture whether and how the relocation behaviour of older parents and their distant children is structured by parents' health issues and the family ties of both parents and children. Each resident of Sweden was identified by a unique identification number that enabled us to link individuals to their family members and across different registers. Annually updated socioeconomic information was derived from the Longitudinal Integration Database for Health Insurance and Labour Market Studies. The analysis was restricted to all noninstitutionalised parents aged 80 years and older and their adult children who lived at least 20 km away in the baseline year. The reason for selecting this age‐group was that many people are dealing with health issues by that age, and it is reasonable to assume that their needs for care are stronger than the needs of their adult children. The 20‐km cut‐off was chosen based on studies on the critical distance threshold with which instrumental support received from children decreases. Such distance might exceed 20 km (Mulder & Van der Meer, 2009) or, in terms of approximate time spent on a journey, 30 min (Checkovich & Stern, 2002). Older people who did not have children and those whose children lived outside Sweden or all within 20 km of the parent's neighbourhood were excluded from the study.

The data enabled us to trace the geographic relocations of parents and children between the years of 2013 and 2016. We observed parent–child dyads that represented the units of analysis across three pooled time periods: 2013 (*t*_0_) – 2014 (*t*_1_) – 2015 (*t*_2_), 2014 (*t*_0_) – 2015 (*t*_1_) – 2016 (*t*_2_), and 2015 (*t*_0_) – 2016 (*t*_1_) – 2017 (*t*_2_). At *t*_0_, we measured baseline characteristics of the study population. We analysed relocations between ends of several pairs of years *t*_0_ and *t*_1_. The year *t*_2_ was required to compute the variable “closeness to death” at *t*_1_. The data for 2017 were used exclusively to create a measure capturing the older parent's closeness to death.

According to our data (Table 1), in 99,785 older woman‐years and 67,306 older man‐years, the individual was childless. In 120,698 mother–child‐years and 33,552 father–child‐years, the parent was already institutionalised at baseline. It was common for older residents of Sweden to have adult children living closer than 20 km. In our data, only 168,108 mother–child dyads and 124,314 father‐child dyads had a child living at least 20 km away at baseline. This is our study population.

**TABLE 1:** Category frequencies for the study population and dependent variable (data in long form)

Categories in a baseline year	Older women		Older men		Categories of the dependent variable	Older women		Older men	
	Frequency	%	Frequency	%		Frequency	%	Frequency	%
Institutionalised	120,698	7.56	33,552	3.41					
Without children	99,785	6.25	67,306	6.85					
With a child living within 20 km	1,208,606	75.67	757,442	77.08					
With a child living more than 20 km away	168,108	10.53	124,314	12.65	No migration	152,707	90.89	115,032	92.59
					Parent moves closer to a child	878	0.52	607	0.49
					Child moves closer to a parent	1,402	0.83	986	0.79
					Parent moves to institutionalised care	7,630	4.54	3,131	2.52
					Parent moves elsewhere	1,604	0.95	1,429	1.15
					Child moves elsewhere	3,786	2.25	3,050	2.46
Total	1,597,197	100	982,614	100		168,007	100	124,235	100

*Note.* We initially planned two additional categories of the dependent variable: both move and end up within 10 km of each other (42 mother–child dyads; 33 father‐child dyads); both relocate but the parent–child distance remains longer than 10 km (59 mother–child dyads; 46 father–child dyads). These categories were excluded from the analysis due to meagre numbers. The final analytic sample consisted of 168,007 mother–child‐years and 124,235 father–‐child‐years.

### 3.2 Dependent variable

The primary outcomes of the study were relocations that resulted in a distance that was less than 10 km between the parent and the adult child. As Gillespie and Mulder (2020) found, moving towards family can be considered a reasonably valid proxy for family‐motivated migration. We considered relocation to within 10 km as convergent because this distance can be travelled in less than 30 min, thereby enabling relatively frequent contact and support (Zhang et al., 2013).

Because residents of Sweden are registered in dwellings clustered within Small Areas for Market Statistics (SAMS), it was possible to identify relocation distances as well as the distances between households of nonresident family members. There are approximately 9,200 SAMS divisions throughout the country, which are based on the subdivision of areas in large municipalities and on election districts in small municipalities. Distance was measured by the Euclidean distance between the geographic centroids of the adult child's and the parent's SAMS‐areas.

Our dependent variable consisted of six categories: (0) no relocation between *t*_0_ and *t*_1_, when neither an older parent nor a distant adult child moves (reference category); (1) an older parent relocates to within 10 km of a child; (2) a child relocates to within 10 km of an older parent; (3) an older parent relocates to a residential care institution; (4) an older parent relocates but the parent–child distance remains longer than 10 km (relocation elsewhere); and (5) a child relocates but the parent–child distance remains longer than 10 km. Summary statistics for the dependent variable are presented in Table 1.

### 3.3 Main explanatory variables

Almost all independent variables were measured at *t*_0_. Our main explanatory variables are indicators of parents' health problems in old age, the gender of the distant child(ren), and parents' and children's family ties.

In the absence of other health measures, closeness to death serves as a proxy for severe health problems (Van der Pers et al., 2015). The measure distinguishes between two categories: the parent did not die within 2 years (the reference category) and they died within 2 years. We are aware that moving could be either a cause or an outcome of death. However, several studies have shown that older movers commonly cite their own poor health as a reason for moving (Choi, 1996). Others have shown that relocation does not bring increases in mortality in old age (Borup, Gallego, & Heffernan, 1979); we therefore consider the potential risk of reverse causality minor.

Because intergenerational geographic proximity is associated with the likelihood of support exchange, older parents' family ties included three categories: at least one child in the same household or neighbourhood, at least one child within 10 km of the neighbourhood, and no children within 10 km (the reference category). Distant children's family ties were operationalised as having at least one sibling within 10 km or not (the reference category).

### 3.4 Control variables

In order to provide an adequate comparison of effect sizes, we controlled for *location‐specific capital* of the target population: presence of a partner in the household, duration of residence, the size of older parents' dwelling, dependent children residing in the household, employment status, duration of residence, and the size of distant children's dwelling. *Sociodemographic characteristics* include older parents' age, educational attainment, income from pension, and migration background as well as the age, educational attainment, employment status, and disposable income of distant children. *The levels of urbanisation for parents' and children's municipalities of residence* were also included.

We distinguished between those who had and did not have a partner in the household (with “living with a partner” as the reference category). Living with dependent children was defined as being registered in the same household as at least one child under age 18 years (with “no dependent children in the household” as the reference category). *Duration of residence* at the current address was calculated using the date of the last registered move within the country and, to facilitate interpretations of extremely low but statistically significant coefficients, we multiplied the numbers of years residing in the current dwelling by 10. *Dwelling size* was based on the dwelling area in square metres for houses and the number of rooms in apartments: a smaller‐size dwelling (less than 90 m^2^ in houses or up to three rooms in the apartments) and a bigger dwelling (90 m^2^ and more or four and more rooms).

We controlled for whether a distant child was registered as employed (including self‐employment) or unemployed (the reference category) in the baseline year. *Age* was measured in years. We distinguished between receiving a pension and disposable income below or above median (with the reference categories pension below median and disposable income below median). Disposable income (in hundred‐thousands of Swedish crowns) was calculated by Statistics Sweden; the few registered negative incomes were recoded to 0. *Educational attainment* was included as a variable with four categories: primary education (the reference category), secondary, postsecondary, and no information. *Immigrant status* identified whether individuals were born in Sweden or another country (the reference category). We applied Eurostat's definition of the level of urbanisation to distinguish between metropolitan areas (the reference category), smaller towns or suburbs, and sparsely populated areas.

Summary statistics for all independent variables are presented in Appendix A.

### 3.5 Analytical strategy

We present the results of two multinomial logistic regression models of migration—the first for mother–child dyads and the second for father‐child dyads. Stratifying the sample by gender enabled us to avoid double counting and correlated outcomes between partners.

We structured the data into long form, such that multiple adult children were nested within their mother or father. The standard errors were adjusted for 56,142 clusters of older mothers and 40,656 clusters of older fathers. Observations were treated as censored after parents relocated closer to a child, moved into an institutionalised care facility, or had a child move closer.

## 4 RESULTS

### 4.1 Descriptive findings

For more than 90% of the parent–child dyads in the study population, intergenerational geographic proximity did not change between 2013 and 2016. Only around 1.3% of older parents and their distant children relocated within 10 km of one another between three pooled time periods of observation, and it was slightly more common among mothers than fathers. One explanation for this relatively low number is that older parents and adult children living far from each other might constitute a select group of less‐family‐oriented individuals, perhaps less willing to exchange support and/or decrease the geographic distance in response to family needs (Vergauwen & Mortelmans, 2020).

Approximately 60% of all convergent moves were made by adult children. When distant children moved closer to parents, the average intergenerational proximity was 2.2 km (*SD* = 2.7) for mothers and 2.4 km (*SD* = 2.8) for fathers. After convergent moves by parents, the average intergenerational proximity was 1.3 km (*SD* = 2.0) for mothers and 1.7 km (*SD* = 2.5) for fathers.

Among dyads where a relocation took place, moves into residential care institutions constituted the largest share of mother–child (4.5%) and father‐child dyads (2.5%). Contrary to our expectations that older people would move into care facilities situated in close proximity to their distant children, additional calculations indicated that for 96.6% of mother–child and 96.5% of father–child dyads, intergenerational geographic distance exceeded 10 km after the parent's institutionalisation. In 2.4% of mother–child dyads and 2.5 of father‐child dyads, parents moved to care facilities close to their children. Only in about 1% of dyads did a child move closer to the newly institutionalised parent. These findings might indicate that institutionalisation and intergenerational proximity moves are two different strategies to fulfil parents' need for care and that they are not commonly used in combination.

### 4.2 Multivariate analyses

**FIGURE 1:**
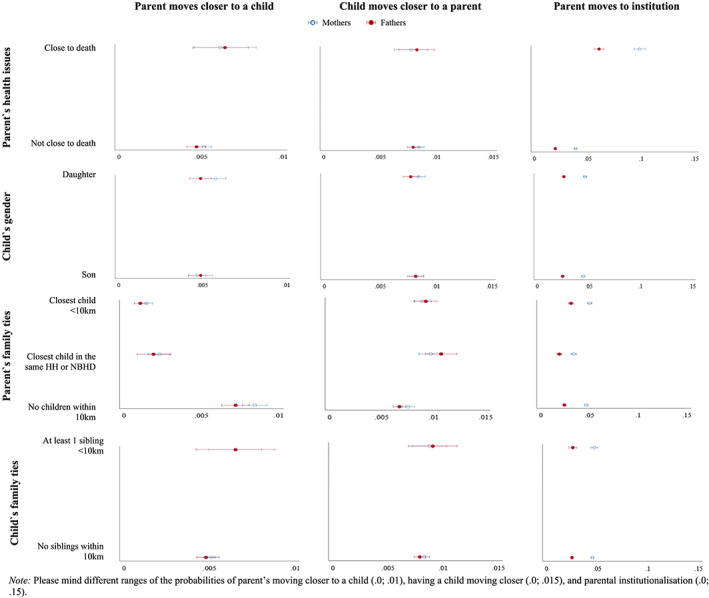
Predicted probability of intergenerational geographical convergence and parent's institutionalisation by the main explanatory variables, estimates, and 95% confidence intervals

Based on predicted probabilities (Figure 1), the findings related to the main explanatory variables and the likelihood of geographic convergence appear similar for older mothers and fathers. The multinomial logistic regression results presented in Tables 2a and 2b lend some support to our hypotheses about the relocation behaviour of older parents and their distant children. *Hypothesis 1a* stated that compared with not moving, parents would be more likely to relocate closer to a child, or have a child move closer, when the parents' health deteriorates compared with when they are in better health. This hypothesis was only partially supported. Fathers' severe health problems increased the likelihood of moving closer to a distant child (*B* = 0.37, *p* < .05). We found a similar effect of health problems on relocation closer to a child for mothers, although the effect was only marginally significant (*B* = 0.26, *p* < .10).

**TABLE 2a:** Estimated multinomial logistic regression coefficients and standard errors (ref: no migration), mothers and nested children

	Parent moves closer to a child		Child moves closer to a parent		Parent moves to institution		Parent moves elsewhere		Child moves elsewhere	
	*B*	*SE*	*B*	*SE*	*B*	*SE*	*B*	*SE*	*B*	*SE*
Closeness to death (ref: did not die within 2 years)										
Died within 2 years	0.262^†^	0.148	−0.012	0.102	1.086^***^	0.037	0.456^***^	0.092	0.072	0.062
Child's gender (ref: son)										
Daughter	0.215^**^	0.071	0.024	0.055	0.048^†^	0.028	0.014	0.054	−0.065^†^	0.035
Parent's family ties (ref: no children within 10 km)										
Closest child coresiding or in the same neighbourhood	−1.329^***^	0.169	0.235^**^	0.072	−0.369^***^	0.049	−0.345^***^	0.083	0.048	0.048
Closest child within 10 km of the neighbourhood	−1.778^***^	0.147	0.156^**^	0.064	0.065^†^	0.036	**−**1.126^***^	0.094	−0.021	0.040
Child's family ties (ref: no siblings within 10 km)										
At least one sibling within 10 km	0.251^*^	0.128	0.066	0.096	0.054	0.046	0.309^***^	0.089	0.139*	0.058
Parent's age	0.021^†^	0.013	0.007	0.008	0.118^***^	0.004	−0.001	0.009	−0.010^†^	0.005
Parent's coresiding partner (ref: living without a partner)										
Living with a partner	−0.294^**^	0.113	0.052	0.065	−0.536^***^	0.051	−0.215^**^	0.080	−0.020	0.041
Parent's education (ref: primary)										
Secondary	0.275^**^	0.104	0.007	0.062	−0.061^†^	0.038	0.098	0.071	0.000	0.040
Postsecondary	0.166	0.140	−0.242^**^	0.088	−0.326^***^	0.057	0.025	0.100	−0.055	0.054
No information	0.418	0.421	−0.130	0.330	−0.337^†^	0.185	0.760*	0.351	0.111	0.202
Parent's pension (ref: pension below median)										
Pension above median	0.168	0.108	0.055	0.067	−0.093^*^	0.042	−0.188^*^	0.078	0.088^*^	0.042
Parent's dwelling size (ref: smaller)										
Bigger	0.296^**^	0.104	0.142^*^	0.065	0.396^***^	0.040	0.277^***^	0.077	−0.038	0.041
Parent's duration of residence in a baseline dwelling	−0.014^***^	0.003	−0.000	0.002	−0.056^***^	0.001	−0.006^**^	0.002	−0.003^**^	0.001
Parent's country of origin (ref: born outside Sweden)										
Born in Sweden	−0.285^*^	0.134	0.015	0.089	−0.047	0.058	−0.299^**^	0.101	−0.087	0.055
Level of urbanisation of parent's place of residence (ref: metropolitan area)										
Smaller town or suburb	−0.338^**^	0.107	−0.230^***^	0.070	0.043	0.043	0.208^*^	0.096	0.079^†^	0.045
Sparsely populated area	−0.406^***^	0.111	−0.193^**^	0.072	0.002	0.045	0.676^***^	0.092	−0.000	0.047
Child's age	−0.025^**^	0.007	−0.019^***^	0.006	0.008^*^	0.003	0.005	0.006	−0.009^*^	0.004
Child's dependent children in the household (ref: no dependent children)										
Living with at least one child	−0.023	0.088	−0.544^***^	0.077	−0.063^†^	0.035	−0.005	0.064	−0.317^***^	0.046
Child's coresiding partner (ref: living without a partner)										
Living with a partner	0.043	0.085	−0.48^8***^	0.065	−0.007	0.032	−0.026	0.062	−0.278^***^	0.040
Child's education (ref: primary)										
Secondary	−0.159	0.147	0.007	0.101	0.073	0.054	−0.265^**^	0.096	−0.007	0.065
Postsecondary	−0.394^**^	0.152	0.013	0.105	0.026	0.056	−0.293^**^	0.100	−0.012	0.067
No information	1.089*	0.533	0.564	0.484	−0.449	0.391	−0.373	0.711	0.288	0.348
Child's income (ref: income below median)										
Income above median	−0.025	0.078	−0.187^**^	0.062	−0.000	0.029	−0.027	0.055	−0.071^†^	0.038
Child's employment status (ref: unemployed)										
Employed	−0.077	0.110	−0.243^**^	0.080	−0.002	0.038	0.082	0.082	−0.069	0.053
Child's dwelling size (ref: smaller)										
Bigger	0.150^†^	0.083	−0.238^***^	0.063	0.009	0.032	−0.036	0.061	0.001	0.039
Childs's duration of residence in a baseline dwelling	−0.012^**^	0.004	−0.050^***^	0.004	−0.003^*^	0.001	−0.002	0.003	−0.058^***^	0.002
Level of urbanisation of child's place of residence (ref: metropolitan area)										
Smaller town or suburb	0.190^*^	0.096	0.226^***^	0.068	−0.019	0.034	0.055	0.064	0.433^***^	0.042
Sparsely populated area	0.307^**^	0.098	0.417^***^	0.069	−0.029	0.036	0.239^***^	0.069	0.603^***^	0.044
Constant	−4.412^***^	1.046	−3.047^***^	0.646	−12.737***	0.347	−4.375^***^	0.732	−1.631^***^	0.410
Model summary										
Wald chi^2^(140)	7,569.35									
Prob > chi^2^	0.0000									
Log pseudolikelihood	−64,961.248									
Pseudo *R* ^2^	0.089									

^†^ *p* < 0.10.

^*^ *p* < 0.05.

^**^ *p* < 0.01.

^***^ *p* < 0.001.

**TABLE 2b:** Estimated multinomial logistic regression coefficients and standard errors (ref: no migration), fathers and nested children

	Parent moves closer to a child		Child moves closer to a parent		Parent moves to institution		Parent moves elsewhere		Child moves elsewhere	
	*B*	*SE*	*B*	*SE*	*B*	*SE*	*B*	*SE*	*B*	*SE*
Closeness to death (ref: did not die within 2 years)										
Died within 2 years	0.375^*^	0.164	0.088	0.106	1.252^***^	0.054	0.432^***^	0.097	−0.064	0.066
Child's gender (ref: son)										
Daughter	−0.000	0.084	−0.055	0.065	0.050	0.044	−0.018	0.056	0.005	0.039
Parent's family ties (ref: no children within 10 km)										
Closest child coresiding or in the same neighbourhood	−1.347^***^	0.291	0.458^***^	0.087	−0.278^***^	0.083	−0.662^***^	0.116	−0.013	0.057
Closest child within 10 km of the neighbourhood	−1.929^***^	0.204	0.316^***^	0.077	0.255^***^	0.059	−1.183^***^	0.119	0.019	0.047
Child's family ties (ref: no siblings within 10 km)										
At least one sibling within 10 km	0.313^†^	0.185	0.144	0.131	0.039	0.086	0.106	0.126	−0.064	0.086
Parent's age	0.001	0.016	0.020^*^	0.010	0.110^***^	0.007	−0.016	0.010	0.004	0.006
Parent's coresiding partner (ref: living without a partner)										
Living with a partner	−0.478^***^	0.119	0.130^†^	0.071	−0.950^***^	0.056	−0.417^***^	0.074	−0.020	0.041
Parent's education (ref: primary)										
Secondary	−0.033	0.144	−0.012	0.077	−0.099	0.062	−0.047	0.084	−0.005	0.046
Postsecondary	−0.030	0.155	−0.088	0.088	−0.373^***^	0.075	−0.091	0.099	−0.010	0.052
No information	0.380	0.453	0.179	0.319	−0.294	0.309	0.648^†^	0.348	0.013	0.204
Parent's pension (ref: pension below median)										
Pension above median	0.101	0.202	−0.164^†^	0.089	−0.153^*^	0.077	−0.351^***^	0.099	−0.145^**^	0.054
Parent's dwelling size (ref: smaller)										
Bigger	0.329^**^	0.127	−0.007	0.073	0.268^***^	0.060	0.148^†^	0.085	0.044	0.045
Parent's duration of residence in a baseline dwelling	−0.153^***^	0.037	0.003	0.020	−0.556^***^	0.022	−0.104^***^	0.023	−0.045^***^	0.012
Parent's country of origin (ref: born outside Sweden)										
Born in Sweden	−0.588^**^	0.183	0.174	0.116	−0.072	0.106	−0.077	0.140	−0.150^*^	0.065
Level of urbanisation of parent's place of residence (ref: metropolitan area)										
Smaller town or suburb	−0.378^*^	0.157	−0.278^***^	0.084	0.009	0.071	0.187^†^	0.111	0.006	0.052
Sparsely populated area	−0.507^**^	0.160	−0.230^**^	0.086	0.033	0.074	0.484^***^	0.105	0.032	0.054
Child's age	−0.019^*^	0.008	−0.035^***^	0.005	0.003	0.004	0.005	0.005	−0.013^***^	0.003
Child's dependent children in the household (ref: no dependent children)										
Living with at least one child	0.095	0.113	−0.568^***^	0.086	−0.022	0.050	−0.012	0.065	−0.293^***^	0.049
Child's coresiding partner (ref: living without a partner)										
Living with a partner	0.087	0.107	−0.433^***^	0.080	−0.015	0.050	0.134^*^	0.067	−0.343^***^	0.047
Child's education (ref: primary)										
Secondary	−0.479^**^	0.164	0.176	0.138	0.012	0.079	0.123	0.118	−0.019	0.078
Postsecondary	−0.680^***^	0.173	0.208	0.144	−0.019	0.083	−0.067	0.123	−0.097	0.081
No information	−0.097	1.035	−13.259^***^	0.239	−0.857	0.756	−13.465^***^	0.214	−0.665	0.679
Child's income (ref: income below median)										
Income above median	−0.133	0.091	−0.294^***^	0.073	0.093^*^	0.045	0.035	0.060	0.002	0.042
Child's employment status (ref: unemployed)										
Employed	0.014	0.147	−0.214^*^	0.103	−0.045	0.065	−0.025	0.099	−0.101	0.064
Child's dwelling size (ref: smaller)										
Bigger	0.018	0.104	−0.225^**^	0.077	−0.024	0.048	−0.107^†^	0.064	−0.120^**^	0.045
Childs's duration of residence in a baseline dwelling	−0.066	0.053	−0.680^***^	0.056	−0.010	0.022	−0.085^**^	0.032	−0.616^***^	0.030
Level of urbanisation of child's place of residence (ref: metropolitan area)										
Smaller town or suburb	0.229^†^	0.123	0.343^***^	0.081	−0.076	0.051	0.061	0.069	0.507^***^	0.047
Sparsely populated area	0.361^**^	0.129	0.559^***^	0.084	−0.003	0.056	0.124^†^	0.075	0.718^***^	0.049
Constant	−2.399^†^	1.383	−3.597^***^	0.782	−11.843^***^	0.548	−2.582^**^	0.869	−2.187^***^	0.482
Model summary										
Wald chi^2^ (140)	11,792.60									
Prob > chi^2^	0.0000									
Log pseudolikelihood	−42,029.401									
Pseudo *R* ^2^	0.0876									

^†^ *p* < 0.10.

^*^ *p* < 0.05.

^**^ *p* < 0.01.

^***^ *p* < 0.001.

Contrary to *Hypothesis 1b*, the effect of poor parental health on children's moves towards parents was nonsignificant. However, in line with *Hypothesis 1c*, compared with no relocation, the effects of health problems were indeed stronger on relocation to institutionalised care (mothers: *B* = 1.09, *p* < .001; and fathers: *B* = 1.25, *p* < .001) than on convergent moves by parents and children (Table 3).

**TABLE 3:** Test for differences in the effects of closeness to death on moving closer to a child, having a child move closer, and institutionalisation

	Moving closer to the child vs. having a child move closer		Moving closer to the child vs. nstitutionalisation		Having a child move closer vs. institutionalisation	
	chi^2^ (1)	Prob > chi^2^	chi^2^ (1)	Prob > chi^2^	chi^2^ (1)	Prob > chi^2^
Mothers	2.34	0.1263	29.59	0.0000	103.36	0.0000
Fathers	2.18	0.1396	25.95	0.0000	98.21	0.0000

With regard to distant children's gender, and in partial support of *Hypothesis 2a*, compared with not moving, older mothers were more likely to move towards daughters than sons (*B* = 0.22, *p* < .01). The results did not point to a relationship between child's gender and father's relocation towards their child, provides partial support for *Hypothesis 2c*, that distant child's gender would be an important predictor for mothers only. Contrary to our expectations that the likelihood of having daughters move closer will be greater than the likelihood of having sons do so (*Hypothesis 2b*), the association between distant child's gender and the likelihood of geographic convergence was found for neither mothers nor for fathers.

Subsequent hypotheses concerned the family ties of older parents. We distinguished between two levels of closer proximity compared with not having children within 10 km: (a) the presence of a child in the same household or neighbourhood and (b) the presence of a child within 10 km of the neighbourhood. The results supported *Hypothesis 3a* and indicated that compared with not moving, older mothers and fathers who had other children living nearby were indeed less likely to move towards the distant child than those who did not have other children living within 10 km. The negative effect of having a child within 10 km of the neighbourhood (mothers: *B* = −1.78, *p* < .001; and fathers: B = −1.93, *p* < .001) was more pronounced than the effect of having a child even closer (mothers: *B* = −1.33, *p* < .001; and fathers: *B* = −1.35, *p* < .001).

**FIGURE 2:**
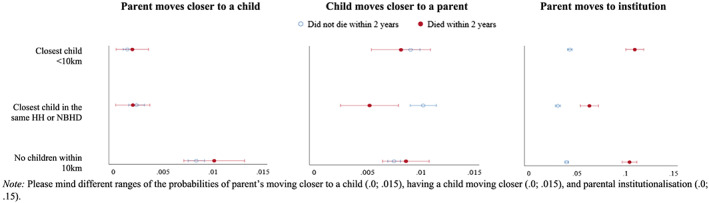
Predicted probability of intergenerational geographical convergence and parent's institutionalisation by the parent's family ties in interaction with closeness to death, estimates, and 95% confidence intervals, mothers

We further hypothesised that there would be a similar effect for the presence of a child nearby on the propensity of institutionalisation in old age (*Hypothesis 3b*). Our results lend only partial support to this hypothesis. Relative to not having any child within 10 km of the neighbourhood, coresiding with an adult child or having a child in the same neighbourhood decreased the likelihood of moving to institutionalised care facilities (mothers: *B* = −0.37, *p* < .001; and fathers: *B* = −0.28, *p* < .001), whereas the presence of a child within 10 km of the parental neighbourhood was positively associated with the propensity of institutionalisation (mothers: *B* = 0.07, *p* < .1; and fathers: *B* = 0.26, *p* < .001). An additional model that included interaction effects between parent's family ties and parental health problems [LR *χ* ^2^(10) = 28.6, *p* = .001] revealed that, for older women, having a child in the same household or neighbourhood decreased the predicted probability of moving to an institution more for those with health issues than for those in better health (see Figure 2—tables with regression coefficients are available upon request). Accounting for similar interaction effects did not improve the model for fathers significantly [LR *χ* ^2^(10) = 9.8, *p* = .463].

We expected that the parent's family ties would be positively associated with the likelihood of having a distant child move closer (*Hypothesis 3c*). Indeed, the presence of at least one child in the same household or neighbourhood (mothers: *B* = 0.24, *p* < .05; and fathers: *B* = 0.46, *p* < .001), as well as within 10 km of the parent's neighbourhood (mothers: *B* = 0.16, *p* < .05; and fathers: *B* = 0.32, *p* < .001), increased the propensity of a distant child's relocation to within 10 km of the parent. The additional model revealed opposing patterns depending on mothers' health problems (Figure 2). When the mother did not have health issues, the predicted probability of a distant child's convergent move was higher if there was an additional child/sibling living in the parental household or neighbourhood (.01) than if no children/siblings were living within 10 km of the parent's neighbourhood (.007). However, when the mother had health issues, the highest predicted probability of the distant child's convergent move was if there were no other children/siblings living within 10 km of the parent's neighbourhood (.009) relative to the presence of an additional child/sibling within the parental neighbourhood (.005) or within the 10‐km radius of the neighbourhood (.008), although confidence intervals were overlapping.

The final set of hypotheses concerned the family ties of distant children. In line with our expectations (*Hypothesis 4a*), older people who had at least two distant children clustered within 10 km of each other were more likely to move closer to them (mothers: *B* = 0.25, *p* < .05; and fathers: *B* = 0.31, *p* < .1) than parents who had a distant child without any siblings living in the 10‐km radius. Contrary to *Hypothesis 4b*, the effect of having at least two distant children living in close proximity to each other on having a distant child move closer was positive but not statistically significant.

The models also showed a positive effect of a distant child's family ties on the likelihood of the mother's relocation elsewhere. Additional descriptive analysis suggests that when parents moved elsewhere and there was a sibling within 10 km of a distant child, the new distance was quite large, indicating that the parent likely chose another destination rather than proximity to that sibling.

A summary of our hypotheses and main results is presented in Table 4.

**TABLE 4:** Summary of hypotheses and research results

Hypothesis	Result
*H1a*: Parents will be more likely to relocate closer to a child or have a child move closer when parents have severe health problems than when they are in better health.	Partly supported. The effect of parent's health issues on moving closer to a distant child was significant for fathers and marginally significant for mothers. There was no similar effect for having a distant child move closer.
*H1b*: The effect of health issues will be stronger for moving closer to a child than for having a child move closer.	Not supported.
*H1c*: The effect of severe health problems will be stronger for relocation to institutionalised care than for geographic convergence.	Supported.
*H2a*: The propensity to migrate towards daughters will be greater than the propensity to move towards sons.	Partly supported. The effect was found for mothers but not fathers.
*H2b*: The propensity to have a daughter move closer will be greater than the propensity to have a son move closer.	Not supported.
*H2c*: The effect of the distant child's gender on the propensity of convergence will be present only for older mothers.	Partly supported. Older mothers were more likely to move closer to daughters than sons, but there was no effect of distant child's gender on the child moving closer.
*H3a*: Older people who already have other children living nearby will be less likely to move towards their distant child than those who do not have children in close proximity.	Supported.
*H3b*: Older people who already have other children living nearby will be less likely to become institutionalised than those who do not have other children in close proximity.	Partly supported. Coresiding with an adult child or having a child in the same neighbourhood decreased the likelihood of moving to institutionalised care facilities, whereas the presence of a child within 10 km of the parental neighbourhood was positively associated with the likelihood of institutionalisation.
*H3c*: Having other children nearby will be associated with a higher likelihood of a distant child moving closer.	Supported.
*H4a*: Older people who have at least two distant children clustered in one location will be more likely to move closer to them than those who have a distant child with no siblings nearby.	Supported.
*H4b*: Older people who have at least two distant children clustered in one location will be less likely to have at least one of these distant children move closer than those who have a distant child with no siblings nearby.	Not supported.

### 4.3 Control variables

Regarding location‐specific capital, parents who lived with partners were less likely to move closer to distant children, elsewhere, or to care institutions. For adult children, having a partner as well as at least one dependent child was associated with a lower likelihood of moving closer to a parent and elsewhere. A similar effect was found for the duration of residence: the longer the parents or children lived in the baseline dwelling, the less likely they were to move to any of the destinations. For older parents, living in a bigger dwelling in a baseline year was associated with an increased likelihood of moving closer to a child, to care facilities, and elsewhere. This might be a strategy to downgrade large housing in light of an increased need for care; however, for mothers, a larger dwelling was also associated with a higher likelihood of having a child move closer. Adult children living in bigger dwellings were less likely to relocate closer to parents or elsewhere (although this result was statistically significant only in the model for older mothers).

For sociodemographic characteristics, parental age was positively and marginally significantly associated with the likelihood of moving closer to a child for mothers, having a child moving closer for fathers, moving to institutionalised care for both, potentially indicating an increase in the older age‐related need for support and the ways to meet this need through informal and formal sources of support. Distant child's age was negatively associated with the likelihood of parents moving closer to this child, having the child moving closer, and having the child moving elsewhere. It was positively associated with the likelihood of institutionalisation for mothers. Better‐educated parents were less likely to move to care facilities. Those with a pension above the median were also less likely to be institutionalised or to move elsewhere. Adult children with primary education were more likely to have a parent moving closer than better‐educated ones. Employed children, as well as those with the income above median, were less likely to move closer to an older parent. Additionally, the fathers of distant children with higher income were more likely to move to institutionalised care. Older parents born in Sweden were less likely to move closer to distant children or elsewhere than foreign‐born individuals (although the effect was significant only for older mothers).

Older parents residing in less‐urban settings in the baseline years were less likely to move closer to distant children and have distant children moving closer than those living in metropolitan areas. Distant children from smaller towns and sparsely populated areas were more likely than those from cities to move closer to their older parents or have parents move closer.

### 4.4 Sensitivity analyses

We performed sensitivity analyses based upon recent findings from Vergauwen and Mortelmans (2020). They showed that children and parents moving into coresidence or closer than 5 km one of another had more frequent support provision compared with longer intergenerational distances following a move, indicating that informal care is perhaps more workable in shorter distances to parents. At the same time, they revealed that in Sweden, children and parents tend to live more than 5 km apart following a move. Accordingly, we ran models with different distance thresholds, where moving closer meant moving within a 5‐ and 15‐kilometre radius of the family member, respectively. The results of the sensitivity analysis did not show substantial differences in the effects of the explanatory variables relative to the models presented in Tables 2a and 2b. We therefore retained the 10‐km radius as a close enough distance, which is also supported by findings of a Swedish study by Johansson (1991). His qualitative results indicated that adult children can guarantee sufficient help even if they live within a short drive from their older parent. Within 10 km of the neighbourhood would indeed be considered a short drive in Sweden.

An additional sensitivity check explored the stability of our models for parents of one or two children and those who have more children. The 95% confidence intervals for the coefficients of these two sets of models were similar. However, for those with three or more children, the (positive) effects of severe health problems and the distant child's family ties on moving closer were not significant. For the parents of one or two children, these effects were slightly stronger relative to the combined models. These differences might be linked to the higher likelihood of having at least one child nearby for parents of many children compared with those who have fewer children (Holmlund, Rainer, & Siedler, 2013). Relocating closer to an older parent or having a parent move closer might be less essential for distant children with many siblings to provide care instead. Those with fewer siblings are more likely to provide support to older parents (Kalmijn & Dykstra, 2006; Stuifbergen, Van Delden, & Dykstra, 2008) and, hence, might be more motivated to move and live closer.

The results of all sensitivity checks are available upon request.

## 5 DISCUSSION AND CONCLUDING REMARKS

Intergenerational geographic convergence can improve adult children's opportunities to care for their older parents. Another option for older parents to receive health‐related care is to move into specialised formal care facilities. We examined the role that parents' health problems play in their relocations—including into institutions—as well as the relocations of their faraway adult children. We were also interested in the effects of distant children's gender and the location of other children on parent/child relocations.

In line with Lin and Rogerson's (1995) and Litwak and Longino's (1987) models, parents' severe health problems were associated with an increase in proximity between children and their parents. The effect of poor parental health on children's moves towards parents was not statistically significant, however. These results are consistent with recent research suggesting that people are more likely to migrate to receive care rather than have family members migrate closer to provide care (Thomas & Dommermuth, 2020). They are also in line with the notion that young adults are the most mobile, followed by the very old (postretirement), with middle‐aged individuals least likely to move, regardless of the distance (Gillespie, 2017).

Although the results regarding the distant child's gender and the likelihood of intergenerational convergence did not wholly support our hypotheses, we found that older mothers were more likely to move closer to daughters than sons. All things considered, when compared with no change in intergenerational proximity, several other characteristics of distant children were associated with moving closer. Broadly, the findings for adult children's characteristics indicated that children moved closer to their older parents in response to their own life circumstances and possibly a need for support. This speculation ties into our findings that (i) having a sibling living near the parent increased the likelihood that a distant child would move closer to their family members and (ii) the attractiveness of this cluster was higher when mothers did not have severe health problems than when they did (the effects were not significant for older fathers). Another explanation might be that this group of distant children moving closer to parents represents the flow of return migration to the place where other family members live to enjoy the benefits of their family's location‐specific capital (DaVanzo, 1981).

The effect of severe health problems was stronger on relocation to institutionalised care than moving closer to distant children. However, coresiding with another adult child, having another child in the same neighbourhood, and living with a partner decreased the likelihood of relocation into care facilities or of moving, more generally. In line with Cantor's (1979, 1991) hierarchical model of support in old age, this suggests that older people might prefer to receive support from their spouse or children than from formal care services. These findings seem especially robust in a Scandinavian context, where informal and formal care are complementary and the role of professional and family care is specialised—informal care is less burdensome and formal care occurs in case of higher care needs.

At the same time, in line with the results of Van der Pers et al. (2015), the presence of a child within 10 km of the parental neighbourhood was positively associated with the propensity of institutionalisation. Because adult children usually support their parents even if they live in the specialised care facilities (Montgomery & Hirshorn, 1991), these findings might indicate that older parents could relocate to an institution nearby if they had had a child in a relatively short distance who could visit.

The findings based on sociodemographic variables showed a negative association between the distant child's education and income and the likelihood of convergence. For parents, education and resources (i.e., pension above the median) were associated with a lower likelihood of institutionalisation. Here, distance might be associated with the provision of financial, rather than other types of support (Bonsang, 2007), and remittances might help subsidise formal care. Moreover, families in which parents or children belong to better‐educated or higher‐income groups might have more opportunities to outsource this type of care, whereas limited resources might signal the need for greater reliance on family and familial proximity (Szebehely & Trydegård, 2012; Ulmanen & Szebehely, 2015).

Swedish register data offer several advantages for studying intergenerational geographic convergence. They enable us to investigate differences between the moves of adult children and their older parents, whose migrations are relatively rare and would be difficult to investigate using survey data. Nevertheless, even with our register data, we encountered small cell sizes in some categories of the dependent variable (e.g., when both parents and children move and end up within 10 km of each other). Another primary advantage of the data is the relative accuracy of the geographic distance between parents and children over time.

One key limitation of our study is the absence of health variables and our use of closeness to death as a proxy. Although studies suggest that relocations are unlikely to contribute to the deterioration of older people's health (Borup et al., 1979; Choi, 1996), reverse causality could be an issue; that is, moving might be a cause or effect of worsening health and subsequent death. Due to restrictions in the data landscape, we were also missing relevant information on parents and children, including homeownership status, the quality of the parent–child relationship, and the actual exchange of support. Prior research suggests that these might be important covariates when considering intergenerational proximity, convergence, and divergence (Rogerson, Burr, & Lin, 1997; Silverstein, 1995; Vergauwen & Mortelmans, 2020).

There are several options for further research on the topic. It would be interesting to focus on labour market outcomes of adult children who moved closer to parents compared with those who moved elsewhere. Including information about parents‐in‐law and their needs for care would allow us to trace how coupled adult children respond to the needs of their own and their partners' parents and to whom they choose to relocate near in the event of competing parental needs. Researchers might also think of using data on other relatives or even neighbours of older people as an approximation of care networks that might deter their migration.

Overall, we extend existing research on intergenerational geographic proximity in several ways. First, we place specific attention on the oldest‐old parents, whose needs for care are likely very high. We provide evidence for a variety of different strategies that older parents might use to resolve their care needs: ageing in place with a child nearby, moving close to a distant child, or receiving formal care from specialised facilities. However, having a child moving closer is not one of these strategies. Second, we account for older parents' severe health problems. Moving in close proximity to children increased when parents had health issues. The attractiveness of parents' locality (amplified when siblings lived there) as a destination for distant children's relocation decreased when parents had health issues. Finally, our results highlight the importance of family in internal migration decisions even in a defamilised context like Sweden—a country known for strong social security provisions that help maintain the independence of citizens of their family members. The role of parents' health issues and role of nonresident family in the relocation behaviour of parents and children might be even more pronounced in less‐generous eldercare models or in more family‐oriented societies.

## CONFLICTS OF INTEREST

The authors declare no conflicts of interest that are relevant to the content of this paper.

## APPENDIX A.

**TABLE A1:** Descriptive statistics, percentage in the sample (or *means*)

	% in sample/*mean*	
	Mothers	Fathers
Main explanatory variables		
Closeness to death		
Did not die within 2 years	91.51	89.43
Died within 2 years	8.49	10.57
Distant child's gender		
Son	49.05	48.59
Daughter	50.95	51.41
Parent's family ties		
Closest child coresiding or in the same neighbourhood	17.10	14.10
Closest child within 10 km of the neighbourhood	29.43	24.78
No children within 10 km	53.47	61.11
Child's family ties		
At least one sibling within 10 km	8.24	5.65
No siblings within 10 km	91.76	94.35
Characteristics of older parents		
Age	*85.2*	*84.4*
Partner in the household		
No partner	73.26	37.86
With partner	26.74	62.14
Education		
Primary	44.52	34.89
Secondary	33.24	34.48
Postsecondary	21.53	29.81
No information	0.71	0.82
Pension		
Below median	69.05	13.12
Above median	30.95	86.88
Dwelling size		
Smaller	60.58	45.76
Bigger	39.42	54.24
Duration of residence	*2.3*	*2.4*
Origin		
Born outside Sweden	9.36	7.96
Born in Sweden	90.64	92.04
Urbanity		
Metropolitan area	22.31	20.57
Smaller town or suburb	39.06	39.31
Sparsely populated area	38.63	40.11
Characteristics of distant children		
Age	*57.2*	*53.4*
Dependent child in the household		
No dependent children in the household	61.38	51.48
At least one dependent child in the household	38.62	48.52
Partner in the household		
No partner	38.03	37.85
With partner	61.97	62.15
Education		
Primary	7.23	6.27
Secondary	32.78	32.97
Postsecondary	59.84	60.66
No information	0.14	0.09
Disposable income		
Below median	42.06	43.78
Above median	57.94	56.22
Employment status		
Unemployed	15.05	10.07
Employed	84.95	89.93
Dwelling size		
Smaller	38.03	38.36
Bigger	61.97	61.64
Duration of residence	*1.3*	*1.2*
Urbanity		
Metropolitan area	40.13	41.37
Smaller town or suburb	33.58	33.74
Sparsely populated area	26.29	24.89
Total	168,007	124,235
